# The impact of interpretive and reductive front-of-pack labels on food choice and willingness to pay

**DOI:** 10.1186/s12966-017-0628-2

**Published:** 2017-12-19

**Authors:** Zenobia Talati, Richard Norman, Simone Pettigrew, Bruce Neal, Bridget Kelly, Helen Dixon, Kylie Ball, Caroline Miller, Trevor Shilton

**Affiliations:** 1 School of Psychology and Speech Pathology, Curtin University, Kent St, Perth, WA 6102 Australia; 2 The George Institute for Global Health, Sydney, NSW 2000 Australia; 3 Early Start, School of Health and Society, University of Wollongong, Wollongong, NSW 2522 Australia; 4 Centre for Behavioural Research in Cancer, Cancer Council Victoria, Melbourne, VIC 3004 Australia; 5 Institute for Physical Activity and Nutrition, School of Exercise and Nutrition Sciences, Deakin University, Geelong, VIC 3220 Australia; 6 South Australian Health and Medical Research Institute, Adelaide, South Australia 5005 Australia; 7 School of Public Health, University of Adelaide, Adelaide, South Australia 5000 Australia; 8 National Heart Foundation, Perth, WA 6008 Australia; 9 School of Public Health, Curtin University, Kent St, Perth, WA 6102 Australia; 10 Department of Epidemiology and Biostatistics, Imperial College London, London, SW7 2AZ UK

**Keywords:** Front-of-pack label, Health star rating, Multiple traffic light, Daily intake guide, Discrete choice, Willingness to pay

## Abstract

**Background:**

This study examined how front-of-pack labels and product healthfulness affect choice and willingness to pay across a range of foods. It was hypothesized that: (i) product choice and (ii) willingness to pay would be more aligned with product healthfulness when healthfulness was expressed through the Health Star Rating, followed by the Multiple Traffic Light, then the Daily Intake Guide, and (iii) the Nutrition Facts Panel would be viewed infrequently.

**Methods:**

Adults and children aged 10+ years (*n* = 2069) completed an online discrete choice task involving mock food packages. A 4 food type (cookies, corn flakes, pizza, yoghurt) × 2 front-of-pack label presence (present, absent) × 3 front-of-pack label type (Daily Intake Guide, Multiple Traffic Light, Health Star Rating) × 3 price (cheap, moderate, expensive) × 3 healthfulness (less healthy, moderately healthy, healthier) design was used. A 30 s time limit was imposed for each choice.

**Results:**

Of the three front-of-pack labels tested, the Health Star Rating produced the largest differences in choices, with 40% (95% CIs: 38%-42%) of respondents selecting the healthier variant, 33% selecting the moderately healthy variant (95% CIs: 31%-35%), and 23% (95% CIs: 21%-24%) selecting the less healthy variant of the four products included in the study. The Multiple Traffic Light led to significant differences in choices between healthier (35%, 95% CIs: 33%-37%) and less healthy products (29%, 95% CIs: 27%-31%), but not moderately healthy products (32%, 95% CIs: 30%-34%). No significant differences in choices were observed by product healthfulness when the Daily Intake Guide was present. Only the Health Star Rating resulted in a significantly greater willingness to pay for healthier versus less healthy products. The Nutrition Facts Panel was viewed for only 7% of all mock packages.

**Conclusions:**

Front-of-pack labels that are more interpretive, such as the Health Star Rating, can be more effective at directing consumers towards healthier choices than reductive front-of-pack labels such as the Daily Intake Guide. The study results provide policy makers with clear guidance on the types of front-of-pack labels that are most likely to achieve positive health outcomes at a population level.

**Electronic supplementary material:**

The online version of this article (10.1186/s12966-017-0628-2) contains supplementary material, which is available to authorized users.

## Background

In many countries, the provision of nutrition information on packaged foods is mandated by governments or voluntarily applied by food manufacturers [1]. The Nutrition Facts Panel (NFP), the most commonly applied form of nutrition information, comprehensively lists the amounts of positive and negative nutrients within a product [2, 3]. In some countries, front-of-pack labels (FoPLs) that present a simplified version of the information contained in the NFP are also provided [4].

Despite the increasing provision of nutrition information on food products, various factors including time pressure, comprehension difficulties, and competing priorities (such as taste, price, promotions, or habit) can prevent people from making use of this information. For example, the NFP is often not used by consumers [5–7], at least partly because it is considered too complex and effortful to interpret [8, 9]. FoPLs attempt to mitigate these barriers through the simplification of nutrition information (to reduce cognitive load) and enhanced prominence on packages (to increase the probability that nutrition value will be factored into food decisions). However, difficulty understanding nutrition information can still persist with FoPLs [10], and even when this barrier is overcome, cognitive biases can prevent people from accurately assessing product healthfulness [11, 12].

An effective FoPL is one that helps consumers distinguish between healthier and less healthy products. Previous research indicates that different FoPLs have varying capacity to achieve this outcome [13, 14]. One common FoPL format that is based on the NFP involves presenting the amounts of key nutrients (such as fat, sugar, and sodium) accompanied by the percent recommended daily intake. This format appears in the Daily Values (used in the US), Reference Intakes (used in the UK), and Daily Intake Guide (DIG: used in Australia and New Zealand) FoPLs. These are known as reductive FoPLs because they reduce the amount of information provided in the NFP but offer little interpretation of this information [15]. As found for the NFP, multiple studies have shown that people find reductive FoPLs difficult and time consuming to interpret [10, 13, 14]. In addition, reductive FoPLs may lead to a positivity bias, whereby the mere presence of the FoPL leads to a more favorable evaluation or increased chance of selecting the product compared to a similar product without a FoPL, regardless of product healthfulness [11, 12, 15–18].

**Fig. 1:**
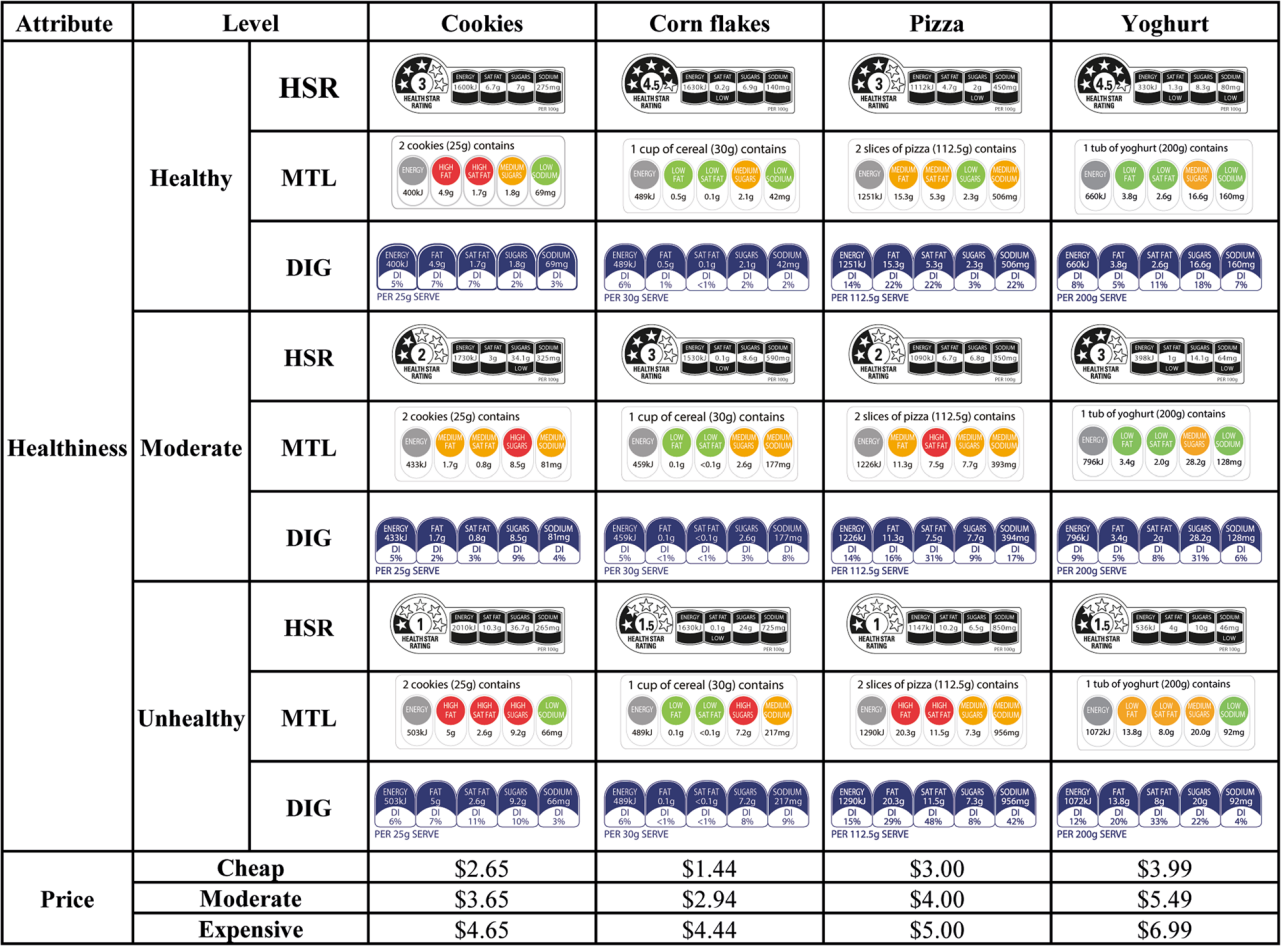
Front-of-pack attributes of study stimuli

In contrast, interpretive FoPLs include features that provide greater evaluation of information contained in the NFP. An example is the use of colors to emphasize whether the level of a particular nutrient is low (green), medium (amber), or high (red) [19, 20]. This occurs in the Multiple Traffic Light (MTL) and the Wheel of Health FoPLs (both from the UK). Interpretive FoPLs may also provide a summary of the overall nutritional profile of a product, such as in the NuVal score (US), the Hannaford Guiding Stars (US), and the Health Star Rating system (HSR). The latter has recently been adopted in Australia and New Zealand and provides both a summary indicator (featuring a star rating that can range from 0.5 to 5 stars) and nutrient specific information (see Fig. 1) [21]. A growing body of research suggests that interpretive FoPLs such as the MTL lead to more accurate impressions of product healthfulness and healthier choices than reductive FoPLs [13, 14]. Interpretive FoPLs with a summary indicator, like the HSR, may be more effective still [12, 22, 23]. There is some (albeit limited) evidence that interpretive FoPLs may also produce a positivity bias [11, 24].

Numerous studies have examined the impact of FoPLs on product selection [11, 15, 17, 19, 25–27], most of which have been conducted online and have focused on the MTL and FoPLs based on a daily intake model (e.g., the DIG) [11, 15, 17, 19, 25, 27]. Most did not include price in their designs and only a few included the HSR or other star-based rating systems [15, 25, 26]. In some of these studies, a single FoPL format appeared on all packages within the choice set [19, 25], which does not necessarily reflect real-world contexts in which manufacturers can choose whether to apply a FoPL to their product. However, widespread use of a single, effective FoPL is likely to have the greatest impact on product selection. For example, supermarket studies using a star-based rating system applied to shelf tags across all products found a shift toward increased purchases of healthier foods [28–30]. Other supermarket studies using the MTL did not observe any shift in purchases, however this may have been due to the label not being applied across all products [31, 32].

Price is important to consider when measuring the impact of FoPLs on choice. Previous research focusing on the MTL indicates that consumers value the ability of this FoPL to communicate information about product healthfulness, which results in higher willingness to pay for healthier versions of foods bearing an MTL. This effect was found when comparing foods with an MTL to those with no FoPL [33] and when comparing foods with a less healthy MTL (more red lights) with a healthier MTL (more green lights) [34]. Research into whether and how other FoPLs affect willingness to pay is lacking, and thus price was included as an independent variable in the present study.

The overall aim of the present study was to examine the effectiveness of three different FoPLs (DIG, MTL, and HSR) in nudging consumers towards healthier choices and away from less healthy choices. These FoPLs range from being reductive (DIG) to more interpretive (MTL and HSR) in nature and would vary in familiarity among the Australian sample in this study. FoPLs are not yet mandatory in Australia and have been used inconsistently to date. The DIG has been in use for over a decade, the HSR was recently adopted in 2014, and the MTL is not widely used. The FoPLs were tested on different foods to assess the generalizability of any effects. Food product variations with a range of healthfulness levels were included to see which FoPLs could both increase consumer choice of healthier products and decrease choice of less healthy products [12]. In light of previous research summarized above, it was hypothesized that:

H1: Product choice would be more aligned with product healthfulness when healthfulness is expressed through the Health Star Rating (HSR), followed by the Multiple Traffic Light (MTL), then the Daily Intake Guide (DIG).

H2: Willingness to pay would be more aligned with product healthfulness when healthfulness is expressed through the Health Star Rating (HSR), followed by the Multiple Traffic Light (MTL), then the Daily Intake Guide (DIG).

H3: Most respondents would not view the NFP.

## Methods

As part of a broader study assessing how varying on-pack nutrition information and price impacts consumers’ food choices, a discrete choice task with a D-efficient design that allowed for estimation of main effects and two-factor interaction effects was created in NGene [35]. The inputs relevant to the present study were: FoPL presence (2 levels), healthfulness (3 levels), and price (3 levels). This design was then replicated across the 4 food types and the 3 FoPL conditions. Further details on the methods employed can be found on the Australian New Zealand Clinical Trials Registry (Trial ID: ACTRN12617000015347).

### Participants

In total, 2069 adult and child respondents were recruited through a large web panel provider (PureProfile). This sample size complies with the recommended minimum of at least 20 respondents per choice set (there were 34 respondents per choice set) [36]. Age, gender, and socioeconomic status (SES) quotas were applied to recruit a diverse sample of Australian consumers (sample profile shown in Table 1). Respondents with a low SES background (i.e., those in Socio-Economic Indexes for Areas (SEIFA) deciles 1 to 4) were deliberately oversampled (49% vs. 40% of the Australian population [37]) to reflect the tendency for these consumers to be less likely to make use of nutrition information [8, 38], have poorer diet quality [39], and experience higher rates of diet-related diseases [40, 41].

**Table 1:** Sample profile (*n* = 2069)

Males (*n* = 1015)			Females (*n* = 1054)		
Age (years)	SES		Age (years)	SES	
	Low^a^	Medium-High		Low^a^	Medium-High
	(*n* = 494)	(*n* = 521)		(*n* = 518)	(*n* = 536)
10–14	69	73	10–14	73	76
15–18	65	68	15–18	69	78
19–25	42	58	19–25	51	53
26–35	64	64	26–35	65	67
36–45	63	64	36–45	65	66
46–55	63	65	46–55	64	65
56–65	64	66	56–65	66	66
65+	64	63	65+	65	65

^a^Low Socio Economic Status category comprised those in SEIFA deciles 1 to 4^28^

As well as being assessed for eligibility based on demographic quotas, respondents were screened for the frequency with which they purchased and consumed the foods featured in the study to ensure the choices were meaningful in the context of their normal diets. To qualify for the study, respondents needed to report purchasing and/or consuming at least two of the products at least occasionally. Data relating to any products never consumed was excluded from analyses. Consent was obtained from adult respondents (and from parents of child respondents) prior to commencing the survey.

### Design

**Fig. 2:**
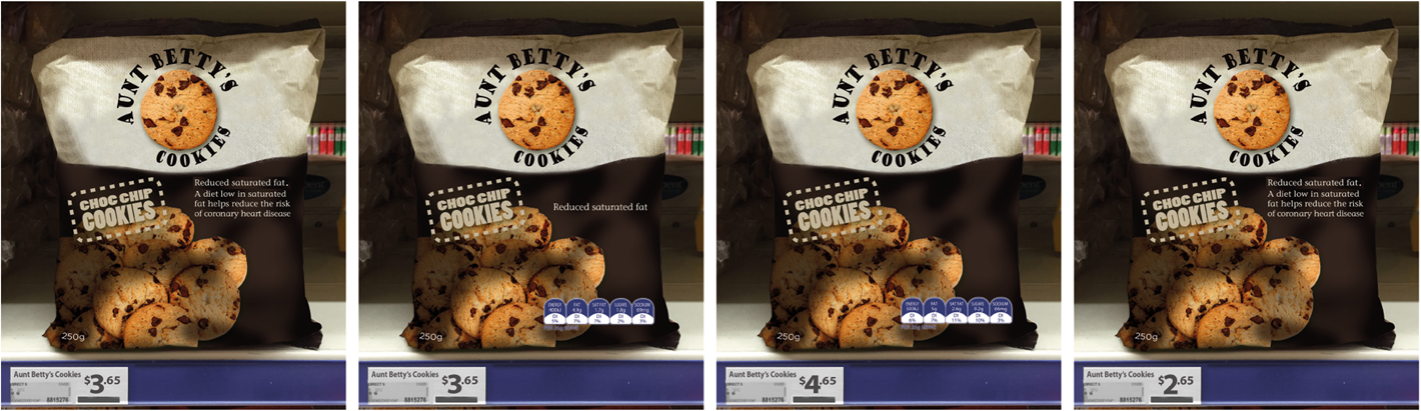
Example choice set

Mock packages were created by a graphic designer to resemble existing food products in the Australian marketplace. Figure 1 shows the levels of the attributes of relevance to the present study that were manipulated on the mock packages. Each respondent was randomized to 1 of the 3 FoPL type conditions for all choice sets and viewed 2 choice sets for each of the 4 food types (no food type was viewed twice in a row). Two packages in each choice set bore a FoPL and 2 packages had no FoPL. Each respondent completed a total of 8 choice sets, with each choice set including four different versions of the same food product with varying levels of healthfulness (see Fig. 2).

### Procedure

The survey (which took approximately 10 min to complete) was completed online via a personal desktop or laptop computer; respondents could not use phones or tablets due to the presentation of choice sets across the screen. Respondents completed a practice choice task with muesli bars to familiarize themselves with the procedure. During the subsequent experimental task, respondents were presented with a row of 4 mock packages and were asked “Given the following options, please select which product you would buy, or if you would not buy any of these products, and click Next”. An option to select “none of the above” replicated the real world context in which consumers can choose not to buy any of the options available [42–44]. Respondents could zoom in on any part of a product image. In addition, they could view the NFP by clicking a link below the image, with this view data recorded to permit analysis of NFP views by FoPL type. To increase the realism of the task and replicate the time pressures often present during food purchase decisions, respondents were given 30 s to make their selection from each choice set, after which time the survey progressed to the next set. This time limit was set based on previous studies [6, 25, 45] and pilot testing. Children (10-17 years of age) completed a similar survey to the adults that had some questions omitted (i.e., food purchasing habits, household income, and education level).

### Analysis

Only choice sets where the respondent picked one of the four product options were included in the analyses (11,244 choice sets). Choice sets were excluded if the respondent timed out (5% of choice sets) or selected the “none of the above” option (18% of choice sets). The attribute levels present in the chosen mock packages were used as dependent variables in the choice analyses. First, the probability of a product being chosen based on its FoPL and level of healthfulness was calculated and plotted for each combination of FoPL and healthfulness. Then, reflecting the binary nature of the dependent variable (each option within a choice set was either selected or not), a series of conditional logistic regression analyses was used to explore the data. The predictors were FoPL type and healthfulness. The conditional logit model is consistent with random utility theory where the utility of each product in the choice set is a function of observed characteristics of that product and a range of unobserved characteristics [46]. Thus, for alternative *i*, the utility function is

$$ {U}_i=V\left(\beta, {X}_i\right)+{\varepsilon}_i, $$

where V is some function of the characteristics of *i*, X_i_ is a vector of the attribute levels of *i*, β is a vector of coefficients and ε_i_ is an error term. Under the conditional logit model, this error term is assumed to follow an independently and identically distributed type 1 extreme value distribution, which yields a probability of selecting alternative *i* of

$$ P\left( Choice=i\right)=\frac{e^{V\left(\beta, {x}_i\right)}}{\sum_j{e}^{V\left(\beta, {x}_i\right)}} $$

where *i* is one alternative among a set of *j* alternatives.

Conditional logistic regressions were run using the *clogit* command in STATA 13, with all data dummy coded. To account for the repeated observations per respondent, the standard errors were adjusted using a clustered sandwich estimator.

As the mock packages varied in price, it was possible to calculate the additional dollar amount that respondents would be willing to pay for a particular level of an attribute (e.g., if respondents were more likely to select an expensive mock package with a DIG over a cheap mock package without a DIG). Regression coefficients were converted and presented as willingness to pay estimates using the *wtp* command in STATA [47]. Separate models were run for each food to explore whether the pattern of results varied by food type.

Statistically significant differences between the different FoPLs (DIG, HSR, MTL) were relative to an omitted base case (i.e., no FoPL control), rather than to each other. Thus, the 95% confidence intervals (CIs) around each coefficient were used to make inferences about meaningful differences between non-omitted levels of each dimension, as recommended [48].

To explore the differential impact of FoPLs on different sub-groups, further willingness to pay analyses were run with respondents separated by gender (males and females), age group (10-17, 18-46, 47+ years), and SES (deciles 1-4 and deciles 5-10) within each individual food type. The 2 adult age groups were created based on the median age among adults in this sample and respondent SES was categorized according to SEIFA deciles [37].

## Results

### Choice probabilities

**Fig. 3:**
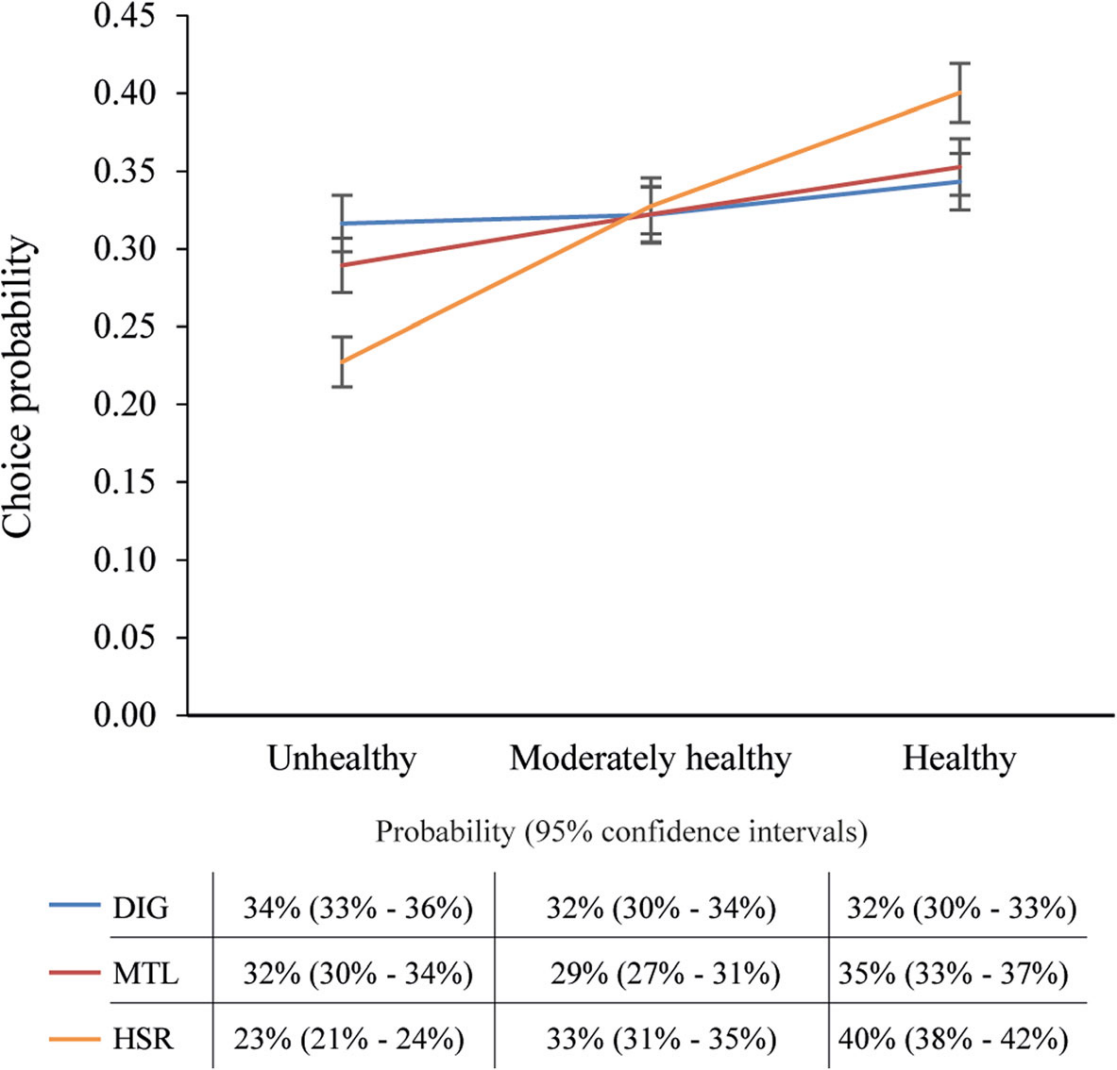
Choice probabilities for the FoPL x healthfulness interaction

Figure 3 shows the frequency with which less healthy, moderately healthy, and healthier products were chosen under the different FoPL conditions. Overlapping confidence intervals across different levels of healthfulness for one type of FoPL suggest the FoPL did not produce marked variations in willingness to pay across different levels of healthfulness. Significantly more respondents chose the healthiest product than a moderately healthy product or a less healthy product in the choice set when an HSR was displayed. When the MTL was present, there was a significant difference between choice of healthier and less healthy products, but not for moderately healthy products. There was no difference in the probability of a healthier, moderately healthy or less healthy product being chosen when the DIG was present on packages. Choice probability for the no FoPL products was around 18%, which is low given that 50% of all mock packages contained no FoPL. This choice frequency did not vary significantly by healthfulness or FoPL condition.

### Willingness to pay

**Fig. 4:**
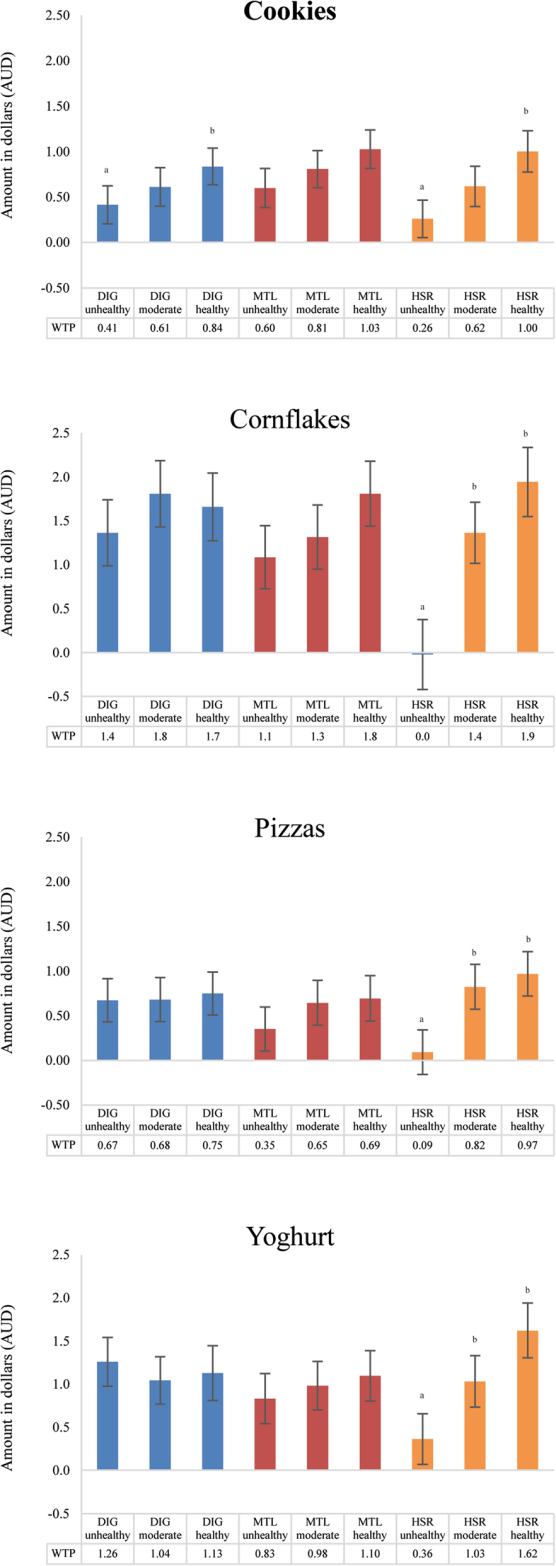
Willingness to pay values by FoPL x healthfulness condition (relative to comparable products with no FoPLs). Note: a significant difference between the levels of healthfulness (within each FoPL) is indicated with different superscript letters

Figure 4 shows the willingness to pay estimates for each FoPL by food type relative to the control condition (i.e., no FoPL). Confidence intervals that do not overlap with the baseline indicate a significant difference in willingness to pay between a particular FoPL x healthfulness condition and the no FoPL condition (demonstrating a positivity bias). Different superscript letters indicate significant differences within FoPL conditions (by healthfulness). Willingness to pay values are presented separately for each food type since each had a different price range. Among products with an HSR FoPL, there was a large, significant increase in willingness to pay for healthier relative to less healthy products (and no overlapping CIs) compared to products with other FoPLs. The presence of an MTL or DIG resulted in no significant difference in willingness to pay across all levels of product healthfulness.

Within each food type, respondents were willing to pay a similar amount for healthier foods across the different FoPL conditions. However, they were less willing to pay for less healthy versions of cornflakes, pizza, and yoghurt with an HSR than a DIG. Of the 3 FoPLs, the DIG produced the smallest variation in willingness to pay across healthfulness levels; the smallest difference between less healthy and healthier product versions was $0.08 for pizzas and the largest was $0.43 for cookies. By comparison, the difference in willingness to pay for product versions of varying healthfulness with an MTL ranged from $0.27 for yoghurt to $0.70 for cornflakes, and the HSR provided the greatest utility with differences in willingness to pay ranging from $0.74 for cookies to $1.94 for cornflakes.

A breakdown of the willingness to pay results according to age, gender, and SES can be found in the Additional file 1. Only 3 differences (i.e., points on the graph where the error bars did not overlap) emerged between demographic categories, none of which indicated any systematic variations in the way different groups of respondents reacted to the predictors.

The NFP view rate was low, with respondents choosing to view the NFP on 7% of mock packages (7% in the DIG condition, 6% in the MTL condition, and 7% in the HSR condition). This low view count may be partially due to the 30 s time limit applied to choices and thus these results may not be directly comparable to other studies.

## Discussion

The present study provides insights into how FoPL type, product healthfulness, and food type combine to influence food choice. Significant differences in choices were observed when the HSR was present on packages, with healthier products being selected the most, followed by moderately healthy products, and then less healthy products. The HSR also produced a significant difference in willingness to pay for healthier versus less healthy product versions across all food types. When the MTL was present, respondents were less likely to choose less healthy foods than healthier foods, suggesting that the MTL was only helpful in assisting consumers differentiate between products at the opposing ends of the healthfulness spectrum. No significant differences in willingness to pay emerged across different levels of healthfulness when the MTL was applied. The DIG performed worst, with no significant differences in choice observed by product healthiness, demonstrating that the DIG did not assist in aligning choice with product healthfulness. Furthermore, respondents were willing to pay a moderately high amount across all levels of healthfulness for cornflakes, pizza, and yoghurt products with a DIG. These results support Hypotheses 1 and 2 in showing that the HSR was most likely to result in choice outcomes and willingness to pay values that were more closely aligned with product healthfulness.

All 3 FoPLs induced at least a slight positivity bias in the present study, as the mere presence of any FoPL increased respondents’ willingness to pay compared to no FoPL (except for the HSR on less healthy cornflakes). Among less healthy foods, the positivity bias was most pronounced for the DIG. This is consistent with previous research showing that people are more likely to choose products with a DIG than products with no FoPL, regardless of their healthfulness [11]. It also aligns with previous research showing that, among less healthy foods, the DIG produces the strongest positivity bias, followed by the MTL and then the HSR [12]. This is an important finding given that a positivity bias in less healthy products could potentially lead to increased energy consumption.

Aside from FoPL type, food type also influenced the healthfulness of respondents’ choices. The most health-conscious choices were made for cornflakes, as evidenced by the larger difference in willingness to pay for healthier relative to less healthy varieties compared to the other foods. This finding is consistent with the idea that cornflakes (and cereals in general) are a category of food that is generally perceived as healthier [49] and for which healthfulness is a primary decision criterion [50]. In reality, cereals show great variation in healthfulness [51, 52]. Children’s cereals in particular tend to be more energy, sugar, and sodium dense and to have lower levels of protein and fiber than adults’ cereal products [52, 53]. As such, applying effective FoPLs to products in this food category could be especially useful in terms of providing consumers with accurate information and addressing incorrect assumptions about product healthfulness.

The other main source of nutrition information included on the mock packages in the present study was the NFP. Respondents had the option to view the NFP, but it was only viewed for 7% of products, supporting Hypothesis 3. This view rate is lower than recorded in previous self-report [6, 54–57] and eye tracking [5, 7] studies, which may have been due to the time limit imposed in the present study.

### Policy implications

The study findings have relevance for policy makers seeking to identify and implement effective front-of-pack nutrition labels. In the first instance, the food industry may exert intense pressure on governments to implement reductive FoPLs in favor of more effective interpretive FoPLs, as evidenced by the €1 billion spent on lobbying against the introduction of the MTL by the European Union [58]. However, gains in public health are unlikely to be made unless decisions are taken in favor of FoPLs that can actually improve consumers’ ability to differentiate products according to their healthfulness. The results of the present study are consistent with those of previous work demonstrating that this improvement is most likely to occur with the application of interpretive FoPLs, and especially those featuring a summary indicator [13, 23]. This growing body of evidence provides support for policy makers attempting to select between the numerous available food label formats. In particular, the present results indicate that the HSR may be worthy of consideration in other nations to assist consumers make healthier food choices.

Second, a related policy implication pertains to the risks associated with allowing nutrition information initiatives to be developed and managed by industry. Not only did the industry-developed label (the DIG) fail to assist consumers select healthier products, it created a positivity bias that could result in higher levels of consumption of unhealthy products than if no FoPL was present. This suggests that the DIG benefits manufacturers rather than consumers, and may actually be a public health liability. This outcome supports the argument that it can be counter-productive to leave public health interventions in the hands of those tasked with optimizing shareholder value [59]. By comparison, the more effective HSR was developed by a committee of representatives from government, public health groups, consumer advocates, and the food industry [60]. The study results show that this approach yielded a FoPL that has the demonstrated ability to facilitate healthier food choices by consumers across a diverse range of age, gender, and SES sub-groups.

### Limitations, strengths, and future research directions

A limitation of the present discrete choice study was that food selections were made in an online context rather than in the real world. Discrete choice experiments are, however, recognized as providing valuable benefits such as greater control over attributes and the ability to efficiently measure the importance of a range of attributes [36]. As such, they are widely used in a range of health promotion contexts including nutrition, vaccination, and tobacco and alcohol control [17, 61–63].

The sample used in the present study was restricted to Australian consumers who were unlikely to be familiar with the MTL. Thus, it is difficult to rule out whether this FoPL would have led to healthier choices if familiarity had been higher. The results are still informative, however, as an effective FoPL would ideally operate at the intuitive level and not require familiarity or an explanation to be used appropriately. Furthermore, if familiarity was a key driving factor, the DIG (with which respondents would have been most familiar) should have performed better in this study.

Another limitation of the study design was the presentation of four very similar mock packages within each choice set that varied primarily on healthfulness (and price) may have resulted in respondents assuming that they were expected to select the healthier product. That considered, the substantial differences observed between each FoPL type demonstrate their varying ability to help consumers distinguish between healthier and less healthy products.

In terms of study strengths, ecological validity was maximized through the use of a variety of foods, time pressure during the choice task, the ability to opt out of choices, realistic product package images, the option to view the NFP, and the inclusion of price. In addition, the large, diverse sample and over-sampling of lower SES respondents provides assurance that the results are relevant to those who may benefit most from more effective food labeling.

Future research should focus on comparing different evaluative FoPLs since there is now strong evidence that they are more effective than reductive FoPLs [13, 14, 64]. Inclusion in future studies of multiple evaluative FoPLs, such as the HSR, the Chilean warning label [65] (which provides information on high levels of negative nutrients only), and the 5 color nutrition label [66] (a summary indicator FoPL that incorporates colors), could reveal more about which FoPL components are most effective. Given that so many different FoPLs are currently being used globally [4], a better understanding of whether FoPLs are country-specific or can be applied in multiple cultural contexts would also be valuable for public policy makers. Finally, there is a shortage of studies conducted in real world shopping contexts, and these are crucial in verifying whether the FoPL effects observed in artificial shopping contexts apply in the real world.

## Conclusions

The results of this study support previous research indicating that the mandated component of food labeling (the NFP) is infrequently used by consumers [6, 8], and hence there is a need for additional food labeling policies that require the consistent provision of more accessible, user-friendly nutrition information. Research to date indicates that interpretive FoPLs are more effective than reductive FoPLs in facilitating healthier choices [13, 14]. The present study extends this work by including the HSR in the analyses. The results support recent studies showing that interpretive FoPLs with a summary indicator may be more effective than other interpretive FoPLs [12, 23]. The HSR increased choice probability and willingness to pay for healthier foods while decreasing these for less healthy foods. The MTL had some impact on choice and willingness to pay (specifically for foods at either end of the healthfulness spectrum), while the DIG had no impact on either outcome variable.

Overall, the findings emphasize the substantial potential of easily understood FoPLs to improve diets at the population level by facilitating increased selection of healthier foods and decreased selection of less healthy foods. The positivity bias produced by the DIG in the current study emphasizes the need for the adoption of a FoPL system that is effective in aiding healthy choices. Of the three FoPLs tested in this study, the HSR appears best suited to this task.

## Additional file

Additional file 1: Willingness to pay by demographic characteristics. (DOCX 55 kb)

## Abbreviations

CI: Confidence intervals
DIG: Daily intake guide
FoPL: Front-of-pack label
HSR: Health star rating
MTL: Multiple traffic light
NFP: Nutrition facts panel
SEIFA: Socio-economic indexes for areas
SES: Socio economic status
