# Supplementary material for: The impact of interpretive and reductive front-of-pack labels on food choice and willingness to pay
Source: Int J Behav Nutr Phys Act. 2017 Dec 19;14:171. doi: 10.1186/s12966-017-0628-2 (PMC5735812; doi:10.1186/s12966-017-0628-2)
Supplement: Additional file 1: — Willingness to pay by demographic characteristics. (DOCX 55 kb) [file 12966_2017_628_MOESM1_ESM.docx]

# Supplementary material

The graphs below show willingness to pay for different foods after the data were split according to socioeconomic status (SES: deciles 1-4, deciles 5-10), gender (males, females) and age (10-17 years, 18-46 years, 46+ years). Just three significant differences were noted. First, females were willing to pay more than males for yoghurt featuring a healthy ($1.00) or moderate HSR ($0.90). Second, children were willing to pay around $0.70 more than adults aged 18 - 46 years for cookies featuring a DIG (across all levels of healthiness). Third, respondents aged 18-46 were willing to pay $1.14 more for yoghurt featuring a healthy DIG.
